# Influence of SiC Polytype on the Thermal Conductivity of SiC Nanofluids: A Critical Review

**DOI:** 10.3390/molecules31050878

**Published:** 2026-03-06

**Authors:** Honorata Osip, Cezary Czosnek

**Affiliations:** AGH University of Krakow, Faculty of Energy and Fuels, al. Mickiewicza 30, 30-059 Kraków, Poland

**Keywords:** nanofluids, thermal conductivity, hybrid nanofluids, silicon carbide, SiC, SiC polytype

## Abstract

Nanofluids are suspensions of nanoparticles in a base fluid. Water, ethylene glycol, engine oil, and others are often used as base fluids, whereas inorganic additives include metals, metal oxides, carbon-based materials, and non-oxide inorganic materials. According to recent research, a small amount of nanoparticles with high thermal conductivity can improve heat transfer in nanofluids. The expectations for nanofluids are increasing, making them the subject of intense research. Current interest is focused on materials that, in addition to high thermal conductivity, also have other favorable features, such as chemical resistance or resistance to high temperatures. Silicon carbide SiC, a material with many advantageous properties, is being considered as a candidate that may meet such expectations. Among the different polymorphs of SiC, the most common are numerous hexagonal α-SiC varieties with anisotropic properties and the only isotropic cubic β-SiC polytype. The latter was reported to have a thermal conductivity of 500 W/mK. The use of nanoparticles from different SiC polytypes in nanofluid studies often leads to incomparable results. Studies on nanofluids prepared from nanoparticles of various silicon carbide polytypes discussed in this article indicate that isotropic β-SiC nanoparticles may be a promising material. When nanofluids for diverse heat transfer applications are prepared, more detailed studies of all the nanoparticles used should be considered.

## 1. Introduction

Liquids, as heat carriers, are important components of heating and cooling systems. For example, cooling is necessary both for the functionality of high-power devices, such as engines, nuclear reactors, and solar energy systems, and for the miniature chips of electronic devices. In recent years, the depletion of oil resources has resulted in an increased demand for renewable energy sources. The attractiveness of solar radiation is that it can generate electricity directly, using photovoltaic (PV) cells, or indirectly by absorbing heat and then converting it into electricity using generators. Photovoltaic modules are attractive to use; however, they have some disadvantages, such as relatively low conversion efficiency. Typical commercial photovoltaic cells are capable of converting only 15–20% of solar radiation into electricity [1,2,3,4,5,6]. The rest is converted into thermal energy, which heats the modules, reducing their efficiency [7,8,9,10]. It is assumed that an increase in the operating temperature of a photovoltaic panel by 1 °C reduces its electrical efficiency by 0.4–0.5% [11]. Cooling mechanisms are necessary to maintain the initial efficiency of PV modules. Pure liquids such as water, ethylene glycol, and engine oil have low thermal conductivity, ranging from 0.1 to 1 W/mK. In contrast, nanofluids have higher conductivity than pure liquids [12]. Nanofluids are suspensions of nanoparticles with sizes below 100 nm dispersed in base fluids. The term “nanofluids” was proposed by Choi and Eastman to describe fluids that, in addition to the base fluid, for example water, engine oil, or ethylene glycol contained an admixture of small amounts of inorganic particles in the nanometer range [13]. Nanoparticle form materials with high thermal conductivity caused a significant increase in the thermal conductivity of the nanofluid compared to that of the base liquid without nanoparticles. Some ceramic materials (i.e., aluminum oxide, silicon nitride, silicon carbide) and metals (i.e., copper, aluminum, silver) have thermal conductivities in the range of 40–350 W/mK and 237–429 W/mK, respectively [14,15]. In turn, synthetic diamond and graphene have even higher thermal conductivity, ranging from 2200 to 5000 W/mK [12,14]. It should be noted that the theoretical and experimental data available in the literature confirm that the thermal conductivity of nanostructures decreases with decreasing nanoparticle size [16,17,18,19]. Anufriev et al. showed that the thermal conductivity of nanostructures is several times lower than that of the bulk material [20]. They also found that the conductivity values scale proportionally to the narrowest dimension of the nanostructures. In the smallest nanostructures, the thermal conductivity reached only 10% of that in the bulk [20], which was still a much higher value than that observed in the base fluids.

The attractiveness of the thermal conductivity of nanofluids makes them the subject of numerous studies, including production techniques, determination of thermal and rheological properties, as well as their specific and potential applications. Nanofluids are used in various energy systems, such as electronic system cooling [21], solar energy systems [1,2,3,5], pipe heat exchangers [22], thermal management [23], nuclear system cooling [24], lubrication [25], energy storage [26], photothermal conversion [27].

Research in this area involves various base liquids with nanoparticles of metals, metal oxides, and non-oxide materials, including some nitrides, carbides, carbon nanotubes, and graphene. The interest extends to mono nanofluids, obtained from a single type of nanoparticles, and hybrid nanofluids, using two or more nanoadditives. Nanofluids containing composite nanoparticle systems are also being investigated. Due to certain limitations of traditional liquids used to produce nanofluids, such as a complicated preparation process or high preparation costs, deep eutectic solvents (DES) are also being investigated as promising base liquids [28,29]. DES are usually obtained by mixing two or more cheap and harmless components under appropriate conditions, and the resulting mixture has a melting point lower than that of any component [30]. In turn, non-oxide nanomaterials such as nanodiamonds, carbon nanotubes (CNTs), or graphene with thermal conductivity and chemical stability higher than those of metal- and metal oxide nanoparticles may be an attractive substitute for the latter. However, due to the high costs of mass production, the use of such nanomaterials as components of nanofluids may be limited [31]. Research is being carried out to overcome the limitations.

The beneficial effect of nanoparticles on the thermal properties of nanofluids is unquestionable. Although heat transfer in nanofluids depends on many factors, the most frequently studied property is their thermal conductivity. In the study by Khouri et al. [32], nanofluids of graphene oxide (GO) nanoparticles (0.1, 0.05 and 0.01 wt%) were prepared in a base fluid composed of deionized water and ethylene glycol. The tests were carried out in a heat exchanger corresponding to an industrial installation at temperatures of 40 °C, 55 °C, 70 °C and 85 °C. The highest increase in TC of 0.380 W/mK was observed for 0.1 wt% GO, while 0.01 wt% GO showed the lowest viscosity of 0.83 mPa, both at 85 °C. Pourpasha et al. [33] investigated nanofluids prepared from transformer oil (TRO) with the addition of multi-walled carbon nanotubes of 0.1 wt% doped with titanium dioxide TiO_2_ (MWCNTs-TiO_2_) and ferrite (ZnFe_2_O_4_) nanoparticles at ratios of 50:50, 75:25, and 25:75. The nanofluid composition containing 0.1 wt% MWCNTs-TiO_2_ nanoparticles has the most significant increase in the thermal conductivity coefficient and volumetric specific heat. The nanofluid containing 0.075 wt% MWCNTs-TiO_2_ and 0.025 wt% ZnFe_2_O_4_ showed the highest improvement in the convective heat transfer coefficient (CHTC) and Nusselt number by 15.38% and 14.4%, respectively, compared to pure TRO. In turn, when the nanoparticle composition was 0.025 wt% MWCNT-TiO_2_ and 0.075 wt% ZnFe_2_O_4_, the nanofluid showed the highest breakdown voltage of 63.52 kV. Aksoy et al. [34] investigated water-based TiO_2_ nanofluids for heat transfer in spray cooling of an aluminum block from 190 to 65 °C. TiO_2_ nanoparticles of 30–50 nm were used at concentrations of 0.05, 0.1, and 0.2 wt%. The authors showed that 0.2 wt% nanofluid reduces the cooling time of the hot block by 25% compared to pure water. They attribute this improvement to the deposition of nanoparticles on the hot surface. At low concentrations, nanoparticles are easily flushed away by the liquid layer, so heat transfer is low. The continuous layer of nanoparticles formed at higher concentrations (0.2 wt%) provides nucleation sites that facilitate heat transfer. This is likely due to agglomeration of nanoparticles near the hot surface during droplet evaporation in the boiling regime. Removing such larger agglomerates by flowing liquid may be difficult. The authors also reported the study [35] on cooling the heated aluminum block from 190 to 100 °C (the boiling regime) by spraying water-based TiO_2_ nanofluids made with nanoparticles of different size ranges. The authors demonstrated that the optimal nanoparticle size, concentration, and deposit thickness can triple the cooling rate. This effect was observed regardless of the size of the nanoparticles. The thickness of the nanoparticle layer was crucial. The thicker the nanoparticle deposition layer, the faster the cooling in the boiling regime.

In turn, Buschmann et al. [36] reported experiments conducted by five independent research teams investigating convective heat transfer in nanofluid flow in pipes, pipe with inserted twisted tape, annular counter flow heat exchanger and coil and plate heat exchangers on the lab scale, as well as full-scale heat transfer apparatus (corresponding to various industrial heat exchangers). The aim of that research was to clarify some common misconceptions regarding the interpretation of convective heat transfer in nanofluids. Water-based nanofluids were prepared from SiO_2_ powders (particle size 12 nm) or by diluting commercially available concentrated suspensions of alumina Al_2_O_3_ (particle size 50 nm, 40 nm and 10 nm), zirconium dioxide ZrO_2_ (particle size 40 nm), magnetite (particle size 60 nm), titanium dioxide TiO_2_ (particle size 85 nm) and multi-walled carbon nanotubes MWCNTs (average length 1.50 μm, and average diameter 9.50 nm). On the basis of the results obtained for dilute nanofluids, the authors concluded that: (i) Newtonian nanofluid flow can be sufficiently described using Nusselt number correlations obtained for single-phase heat transfer liquids such as water when thermophysical properties of nanofluid are utilized. No anomalous phenomena involved in the thermal conduction-based and forced convection heat transfer of nanofluids were observed. In most cases, the Newtonian nanofluids can be treated as homogeneous fluids; (ii) the heat transfer improvement provided by nanofluids equals the increase in thermal conductivity of the nanofluids compared to base fluid when comparing similar thermodynamical and fluid mechanical flows. This was observed regardless of nanoparticle concentration, and the size or material; (iii) the above statements were true for several relevant industrial heat transfer apparatus.

Pugalenthi et al. [37] investigated nanofluids prepared from graphene, SiC, Al_2_O_3_ and SiO_2_ with distilled water (DW) for applications in photovoltaic systems. SiC-based nanofluids showed improved stability. The highest thermal conductivity improvement of 28.03% was achieved for SiC/water nanofluid at a 0.02 vol% nanoparticle concentration compared to pure water. For thermal storage purposes, the authors also studied the thermal properties of a mixture of SiC nanoparticles (0.2 vol%) dispersed in lauric acid phase change material (PCM). The TC of such a system was increased by 29.8% compared to the PCM without nanoparticles.

Heat transfer in nanofluids depends on many factors, such as the type of nanoparticles, their thermal conductivity, grain shape and size, and the content of nanoparticles in the base fluid [38,39]. Properties such as stability, viscosity, density, and thermal diffusivity of nanofluids are also important [40]. New review articles have recently been published that discuss these factors in detail [41,42]. The complexity of the factors often makes it difficult to compare the studies from different research groups, even when chemically identical components were used. Another factor that makes comparison of studies difficult may be the interchangeable use of nanoparticles of different polymorphic varieties of the material with anisotropic or isotropic properties. Model-based theoretical studies, combined with the use of numerical and statistical methods, can facilitate the study of heat transfer processes in nanofluids [43]. Moreover, the use of nanoparticles of materials with well-characterized thermal properties may also be helpful in comparative studies.

**Figure 1:**
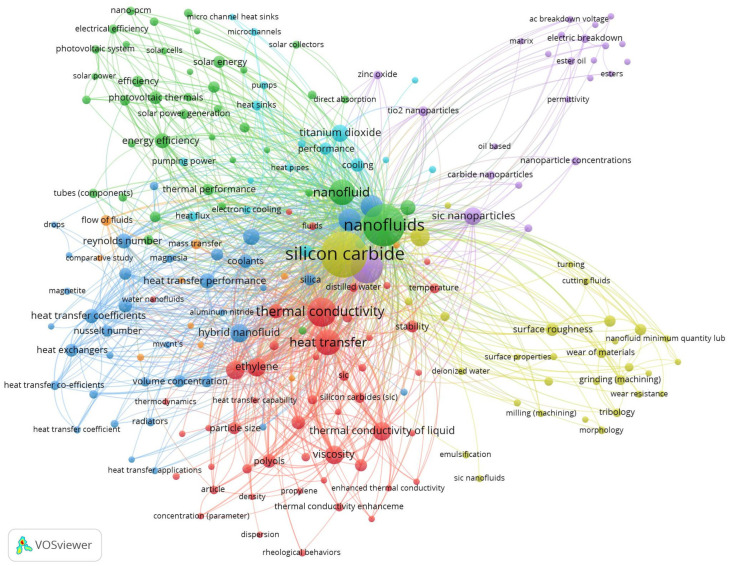
Co-occurrence and interconnections of keywords related to nanofluids and silicon carbide appearing in scientific publications.

SiC nanoparticles, due to their high thermal conductivity as well as chemical and thermal resistance, have often been investigated as an attractive component of nanofluids. Figure 1 shows a network visualization of keyword co-occurrences created by VOSviewer software (Version 1.6.20) based on data obtained from the Scopus database. For this purpose, a search of the Scopus database was performed using the built-in advanced search interface. The following search query was applied to the title of the article, the abstract, and the author’s keywords: “nanofluids” AND “silicon carbide”. The publication period was limited to 2015–2025. Only peer-reviewed research and review articles published in English-language journals were selected, resulting in 252 records. The most common keywords in the literature on <nanofluids AND silicon carbide> are “nanofluids”/“nanofluid” and “silicon carbide” appearing in 264 and 235 publications, respectively. Frequently used keywords are also “nanoparticles” (112), “thermal conductivity” (91), “heat transfer” (74), and “hybrid nanofluids” (37). The occurrence of these latter keywords indicates that nanoparticles are of great interest in research on enhancing the thermal properties of fluids used in various heat transfer systems.

Despite intensive research on the use of SiC nanoparticles in the preparation of nanofluids, the results obtained seem to be incomparable. To our knowledge, there is poor information in the nanofluid literature that considers the thermal properties of silicon carbide in the context of its specific crystal structure. The novelty of this work is the demonstration, based on literature data, of a close correlation between the thermal properties and the crystal structure of SiC polytypes in the context of their application in nanofluids. Data from the literature lead to the conclusion that the structure of the starting material has a significant influence on the heat transfer in nanofluids, especially if the material can crystallize in different polymorphic forms, such as SiC. Therefore, a detailed characterization of the nanoparticles used to prepare nanofluids is absolutely necessary. This paper reviews and discusses the latest reports on nanofluids prepared with SiC nanoparticles, mainly in terms of nanofluid thermal conductivity enhancement. Studies on the use of SiC hybrids with other types of nanoparticles for nanofluid production are reviewed. Last but not least, this paper highlights the influence of the crystal structure and anisotropy of silicon carbide polytypes on the thermal conductivity of SiC nanofluids.

## 2. Silicon Carbide Structural Properties

**Figure 2:**
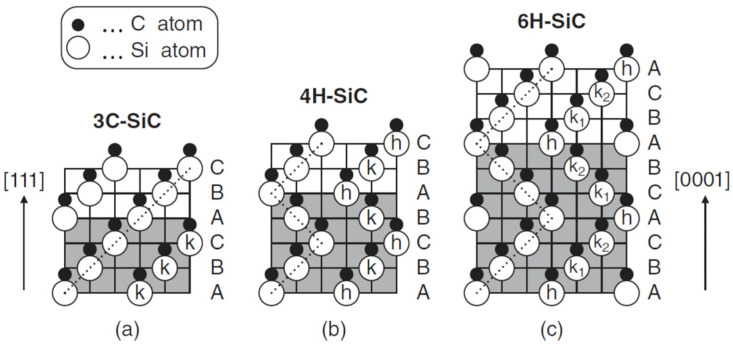
Schematic structures of popular SiC polytypes; (**a**) 3C SiC, (**b**) 4H SiC, and (**c**) 6H SiC. Open and closed circles denote Si and C atoms, respectively [53], with permission from Wiley and Sons.

**Figure 3:**
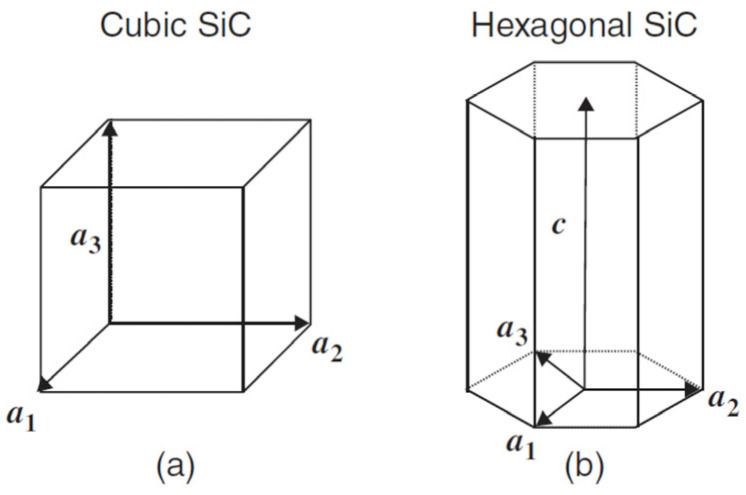
Primitive cells and fundamental translation vectors of (**a**) cubic 3C SiC and (**b**) hexagonal SiC [53], with permission from Wiley and Sons.

Due to the differences in the reported thermal conductivity of SiC nanofluids, it is worth providing some information about silicon carbide and the most important differences between polytypes. In particular, there are several reports on the thermal conductivity of SiC that contain conflicting data on the values of individual structural varieties [44,45,46]. Silicon carbide SiC is a semiconducting ceramic material with exceptionally attractive properties, such as very good thermal conductivity, high strength, and resistance to high temperatures and chemical corrosion. Silicon carbide can exist in the form of β-SiC and α-SiC polymorphs. The β-SiC polytype is also known as a low-temperature polytype (production temperature < 1700 °C), while the α-SiC polytypes are known as high-temperature polytypes (production temperature > 1700 °C). Annealing β-SiC polytype at temperatures above 1800 °C favors its transformation into polytype α-SiC. By neutron irradiation, α ⟶ β transformation can be achieved at lower temperatures, although the process is then limited [47]. The only β-SiC polytype has a cubic structure, while numerous α-SiC polytypes covering more than 200 crystal polymorphs have hexagonal or rhombohedral structures [48,49]. According to Ramsdell notation, the β-SiC polytype is named 3C SiC, while the α-SiC polytypes in this notation are named by the letter H or R corresponding to hexagonal or rhombohedral symmetry, respectively [50,51,52]. The number given before the symmetry letter indicates the number of SiC layers in the stacking order. The best known and most frequently studied polytypes are 3C SiC, 4H SiC, and 6H SiC. Their schematic structures are illustrated in Figure 2, while Figure 3 shows the primitive cells and fundamental translation vectors of these polytypes.

Table 1 shows the lattice constants of most popular silicon carbide polytypes, 3C SiC, 4H SiC and 6H SiC, measured at room temperature.

The traditional method of SiC production is the Acheson process, which is used most frequently in industrial manufacturing [48]. This high-temperature process produces silicon carbide mainly with a hexagonal structure (α-SiC) in the form of large crystals, and grinding is necessary to obtain nanometer-sized particles. The advantage of mechanical methods is a relatively simple production process, but the disadvantage is the reduced sphericity of the resulting nanoparticles [54].

The possibility of producing 6H SiC (α-SiC) nanoparticles at low temperatures using a magnesium catalyst was also reported [55]. Due to the simplicity of controlling the production process, CVD gas phase synthesis [56], plasma synthesis [57], aerosol synthesis [58], the sol-gel method or microwave synthesis [59] are more useful for the direct fabrication of β-SiC nanoparticles [60]. Our group also reported research on the preparation of nanocrystalline cubic silicon carbide β-SiC by the aerosol-assisted synthesis method from liquid organosilicon precursors [57,58]. The powders obtained by this method possess a spheroidal morphology and particle sizes ranging from several to several hundred nanometers.

Thermal conductivity tests were carried out for the most popular hexagonal polytypes 4H SiC and 6H SiC doped with various elements. Table 2 presents the thermal conductivity results of the most frequently tested SiC polytypes reported in the literature. The results for both tested hexagonal SiC polytypes presented there indicate the anisotropic nature of the conductivity in these polytypes. Regardless of the polytype, 4H SiC or 6H SiC, the TC measured in the direction parallel to the C axis (see Figure 3) is lower than that measured perpendicular to the axis. In turn, doping the hexagonal polytype 4H SiC causes a change in thermal conductivity depending on the dopant used.

For example, Wei et al. investigated the dependence of the thermal conductivity of 4H SiC crystals on the type of dopants [45]. They showed that the thermal conductivity of nitrogen-doped 4H SiC at room temperature was 280 W/mK when measured along the C-axis, while the thermal conductivity of vanadium-doped 4H SiC under the same conditions was approximately 347 W/mK. Cheng et al. measured the thermal conductivity of boron- or nitrogen-doped epitaxial 6H SiC and monocrystalline porous 6H SiC below room temperature probed by time-domain thermoreflectance (TDTR) [63]. They observed an order of magnitude reduction in the measured thermal conductivity at low temperatures for porous 6H SiC compared to epitaxial 6H SiC. Such results seem to suggest that it is possible to tune the thermal properties of silicon carbide by doping it with other elements. However, due to the scarcity of experimental data in the available literature, it is difficult to express such general conclusions.

Cheng’s group investigated the thermal conductivity of the cubic β-SiC polytype. The results showed that TC exceeds 500 W/mK at room temperature in high-quality wafer-scale cubic silicon carbide 3C SiC crystals obtained by low-temperature chemical vapor deposition [63]. The authors found that the thermal conductivity of this polytype is 50% higher than the structurally more complex commercially available hexagonal polytype 6H SiC and >50% higher than the conductivity of polytype 3C SiC previously reported in the literature. Unlike the anisotropic thermal conductivity of structurally more complex α-SiC polytypes, the thermal conductivity of the cubic β-SiC polytype is isotropic.

Few experimental studies have investigated the anisotropy of thermal conductivity in SiC polytypes. Due to measurement difficulties, the anisotropy of thermal conductivity of hexagonal silicon carbide polytypes was often ignored in previous experimental works [45,46]. Burgemaister et al. reported an experimental work on the thermal conductivity of 6H SiC crystals measured in the 300–500 K range by radiation thermometry [62]. They experimentally confirmed the anisotropy of the thermal conductivity of the hexagonal SiC polytype. When measured perpendicular to the hexagonal planes (parallel to the c-axis), the thermal conductivity of 6H SiC was approximately 30% lower than the thermal conductivity measured parallel to the hexagonal planes (perpendicular to the c-axis). These observations were essentially confirmed by Qian et al. who used a modern measurement technique [44]. The authors used the time-domain thermoreflectance (TDTR) method to measure both the in-plane and the cross-plane thermal conductivity of three SiC single crystals, N-doped 4H SiC, unintentionally doped 4H SiC, and V-doped 6H SiC, over a temperature range of 250 to 450 K. The anisotropy of thermal conductivity was found in all tested hexagonal SiC samples; the cross-plane was 40% lower than that measured in the plane. The measurements also showed that 4H SiC had higher thermal conductivity than 6H SiC. Changes in the thermal conductivity of SiC polytypes may result from differences in their anisotropy and the presence of impurities in the SiC material or its intentional doping with specific elements.

Differences in the thermal conductivity of SiC-produced nanofluids may be related, among other things, to the polytypes used and the method of preparing the nanoparticles. The selection of a SiC polytype that is suitable for the production of nanofluids with optimal properties requires more extensive research on the properties of these nanoparticles. According to one of the latest studies, the isotropic β-SiC polytype with thermal conductivity even higher than α-SiC polytypes may be a good candidate for applications in nanofluids [61]. The lower production temperature of β-SiC and the possibility of obtaining spherical nanoparticles directly in the synthesis process indicate the advantage of the beta polytype, also due to production costs, relatively simple equipment, and thermal properties. However, further research is needed to determine the influence of nanoparticle-related factors on heat transfer in nanofluids and to satisfactorily explain the mechanisms governing this process.

## 3. Nanofluids Preparation and Properties

Generally, two methods are used to produce nanofluids, namely the one-stage method and the two-stage method. The one-stage procedure was most often used to produce nanofluids containing metallic nanoparticles. The production of nanofluid using this method involves the direct condensation of metal vapors into nanoparticles by contact with a flowing base liquid [64,65,66]. An important advantage of the one-stage method is the relatively low agglomeration of nanoparticles, which has a positive effect on the stability of nanofluids [67,68]. The one-step method can only be used for liquids with low vapor pressure, such as ethylene glycol, which limits the area of application of this method [69].

In the two-stage method, powders in the form of nanoparticles, nanotubes, or nanofibers are first produced by various methods, such as ball milling, laser ablation, chemical vapor deposition CVD, spray pyrolysis, sol-gel, etc. In the next step, the nanopowder is placed in the base fluid and dispersed using mechanical or magnetic stirrers. Since nanoparticles usually tend to agglomerate, it is necessary to use ultrasonic processors [65,70]. Dispersants are often added to increase the stability of nanofluids. This method allows the use of commercially available nanoparticles, which is very useful for the production of nanofluids on a large scale [70]. The properties of nanofluids are significantly influenced by both the type of base fluid and the size and composition of the nanoparticles. Ambreen and Kim reported a comprehensive review on the particle size-dependent thermal conductivity of nanofluids under various conditions [71]. The impact of particle concentration and size on thermal conductivity was systematically analyzed. Several discrepancies were found in the results of the collected research, which resulted from different techniques used to produce nanofluids or to measure thermal conductivity, and from the lack of description of the shape of particles and the size distribution of nanoparticles in the liquid. Furthermore, there was no information on the measurement temperature, type of surfactant, and pH of the system. The authors noted that measurement techniques may be a source of inadequate interpretation of the effect of nanoparticle size on thermal conductivity since most researchers determine dry particle sizes to predict the size of nanoparticles in solution, while particles in the liquid environment may become agglomerated during measurement.

To fully exploit the thermal properties of silicon carbide in heat transfer, many researchers have investigated various nanofluid systems, such as liquid/SiC nanoparticles (mono nanofluids), liquid/SiC nanoparticles with another nanoadditive (hybrid nanofluids), and mixtures of different base liquids with SiC. Attempts have also been made to test nanofluids produced with SiC in simulated laboratory heat transfer systems.

## 4. Mono Nanofluids

Mono nanofluids are dispersions of one type of solid nanosized particles in a base fluid [72]. Several studies on thermal conductivity enhancement of SiC mono nanofluids are summarized in Table 3. Senthilkumar et al. investigated the thermal conductivity of water-based SiC and cryoSiC (cryogenically treated SiC) nanofluids at different temperatures from 30 °C to 50 °C with particle contents of 0.1, 0.2 and 0.3 wt% [73]. They concluded that deep cryogenic treatment results in a drastic improvement in the thermal conductivity of SiC nanopowder by 45%. The thermal conductivity of the SiC/water and CryoSiC/water nanofluids increased by 13.22% and 17.56%, respectively, compared to the base fluid. Li and Zou found that water-ethylene glycol mixture-based SiC nanofluid (SiC/W-EG) with 1.0 vol% SiC nanoparticles showed a 33.84% increase in thermal conductivity compared to the initial base mixture [74]. Nikkam et al. investigated nanofluids produced using both, cubic β-SiC and hexagonal α-SiC nanoparticles (9 wt%) in a water (W) and in a water-ethylene glycol (W-EG) mixture (50/50 wt%) to investigate thermophysical properties, including thermal conductivity and viscosity of these nanofluids [75,76]. The thermal conductivity of the nanofluid with α-SiC nanoparticles was improved by 20% compared to the base liquid, and the viscosity increased by only 14%. The authors showed that nanofluids containing α-SiC nanoparticles have higher thermal conductivity than those produced with β-SiC, attributing this to the influence of the crystal structure. However, they noted that this effect may also be due to the low purity of the nanoparticles used [77]. In fact, all α-SiC nanoparticles used in that study contained only the hexagonal SiC phase, while β1-SiC nanoparticles contained a small amount of α-SiC phase and β2-SiC nanoparticles contained an admixture of the crystalline Si phase, as confirmed by XRD [76]. The higher thermal conductivity of α-SiC nanofluids compared to β-SiC nanofluids is most likely due to both the presence of impurities in β-SiC nanoparticles and the larger size of α-SiC nanoparticles. The nanoparticle size estimated from SEM micrographs was (115 ± 35) nm and (85 ± 20) nm for α1-SiC and α2-SiC, respectively, versus (60 ± 10) nm and (30 ± 10) nm for β1-SiC and β2-SiC, respectively [76]. It has been theoretically and experimentally confirmed that thermal conductivity tends to decrease with decreasing nanomaterial size [16].

Vallejo et al. investigated nanofluids based on the mixture of propylene glycol (PG) and water (PG:W = 30:70), consisting of dispersions at 1 and 2 wt% concentrations of α-SiC and β-SiC nanopowders containing mainly 6H and 3C polytypes, respectively [78]. By evaluation of the Zeta potential, the authors confirmed that nanofluids demonstrated moderately stable dispersions. The authors showed that the SiC crystal structure plays a noticeable role in improving the thermal conductivity of nanofluids. Temperature-independent TC increases up to 4% and 11–12% were obtained for 2% α-SiC and β-SiC nanofluids, respectively. The authors concluded that cubic, β-SiC with a less complex crystal structure improves the thermal conductivity of the nanofluid to a greater extent than SiC with a more complex crystal structure (hexagonal, α-SiC). These conclusions are contrary to those presented by Nikkam et al. [75]. However, in light of new research [61], the β-SiC phase has a higher thermal conductivity than the α-SiC phase.

Akilu et al. investigated ethylene glycol and propylene glycol based β-SiC nanofluids at temperatures between 25 °C and 80 °C [79]. In this study, a greater temperature-dependent increase in the thermal conductivity of ethylene glycol/β-SiC nanofluid compared to propylene glycol/β-SiC nanofluid (14.6% vs. 4.8%, respectively) was observed. The authors attributed this observation to the higher viscosity of propylene glycol, which slows down the Brownian motion of β-SiC nanoparticles in the base fluid (propylene glycol), resulting in lower thermal conductivity. Srinivas and Srinivas comparatively tested SiC nanofluids prepared by dispersing different volume concentrations of SiC nanoparticles (in the range of 0.25 to 2 vol%) in various base fluids: engine oil, heating oil, or transformer oil [84]. The study showed that the thermal conductivity of all nanofluids tested increased with increasing nanoparticle concentration and temperature. In turn, Ajeeb and Murshed investigated the thermophysical properties of SiC nanofluids (particle concentrations from 0.01 to 0.05 vol%) in a mixture of ethylene glycol (EG) and water (W) [85]. They observed the Newtonian rheological behavior of nanofluids and estimated that the maximum increases in viscosity and density of the nanofluid containing 0.05 vol% of nanoparticles were 5.2% and 0.3%, respectively. This nanofluid presented a maximal increase in thermal conductivity of 4%. Ezekwem and Dare investigated the effect of nanoparticle concentration and temperature on the dynamic viscosity of SiC nanofluids prepared by dispersing SiC nanoparticles in ethylene glycol (EG) and water (W) [83,86]. These nanofluids showed an increase in viscosity with an increase in the volume concentration of the nanoparticles. For the 0.5 vol% and 5 vol% SiC/W nanofluids, the viscosity ratio was 1.023 and 1.435 times that of the base fluid. On the contrary, the viscosity of the nanofluids was reduced by increasing the temperature in comparison to that of their base fluids. Huminic et al. investigated the effect of temperature and weight concentration on the thermophysical and heat transfer characteristics of 3C SiC/water nanofluids in a temperature range of 20 °C to 50 °C for two weight concentrations, 0.5 and 1.0 wt% [80]. The result showed that the TC increases by 17.62% with increasing temperature (by 50 °C) and an optimal particle concentration of 1.0 wt%.

## 5. Hybrid Nanofluids

Hybrid nanofluids are produced by simultaneously dispersing two or more component nanoparticles in a base fluid. The purpose of preparing hybrid nanofluids is to improve the thermal properties of the nanofluids and thus increase the heat transfer rate, although the interests of researchers are not limited to this type of nanofluids. Nanoparticles are promising materials for modifying the tribological properties of synthetic liquid lubricants. Mousavi et al. [25] synthesized a Cu/TiO_2_/MnO_2_-doped GO nanocomposite, which was introduced at three different concentrations as a nanoadditive to the prepared synthetic biodegradable polyalphaolefin (PAO) oil, with the total nanoadditive content being 0.3%. This resulted in a synthetic lubricant with exceptional physicochemical properties, particularly in terms of durability and high performance. The authors found that the addition of the nanocomposite resulted in a significant increase in the viscosity index by as much as 19%. The optimal additive content led to an improvement in flash point and pour point by approximately 5% and 24%, respectively. On the other hand, nanocomposites showed a maximum anti-wear feature of approximately 46%.

Hybrid nanofluids are believed to have a thermal conductivity higher than that of mono nanofluids, which is due to the synergistic effect [87,88,89,90]. There are also studies showing that some hybrid nanofluids have a lower thermal conductivity than mono nanofluids [89]. Other studies indicate a variable effect on thermal conductivity as the proportion of the nanoparticle mixture increases [89,91]. Hybrid nanofluids were also highlighted as being often more stable, having better rheological properties, and fewer pipe systems were clogged [40,41,92]. Further research is necessary to resolve such inconsistencies.

Huang et al. investigated hybrid nanofluids containing thermal oil and silicon carbide-multiwalled carbon nanotubes (SiC-MWCNTs) under different conditions in terms of stability, thermal conductivity, and viscosity [93]. Such hybrid nanofluids are expected to acquire properties that are not possessed by a single component. In this study, the average size of the SiC nanoparticles tested was 40 nm, and the average size of the MWCNTs was 20 nm. The authors found that the thermal conductivity of the hybrid nanofluid with a SiC-MWCNT concentration of 1.00 vol% increased by 22.58% at 80 °C, while the stability of the nanofluid was maintained up to a concentration of 3.00 wt%. Taking into account the influence of nanoparticle concentration on the viscosity of the nanofluid and its thermal conductivity, the authors concluded that the optimal concentration of SiC-MWCNTs nanoparticles in the base-fluid should be 0.10 vol%.

Several studies on the thermal conductivity enhancement of hybrid SiC-containing nanofluids are summarized in Table 4.

Kim et al. investigated the photothermal conversion efficiency and solar energy absorption of water-based hybrid silicon carbide-indium tin oxide (SiC-ITO/W) nanofluids to develop a working agent in a direct absorption solar collector (DASC) [97]. They found that the photothermal conversion efficiency of the SiC-ITO hybrid nanofluid is higher than that achieved when the SiC and ITO nanoparticles were used independently. In another work, Li et al. studied ethylene glycol-based SiC-MWCNTs nanofluids to prepare hybrid nanofluids with enhanced stability and high solar thermal conversion efficiency for DASC applications [98]. The authors proved that such hybrid nanofluids had very good stability and demonstrated an excellent ability to absorb solar radiation in both the visible and near-infrared regions (200–1100 nm). The thermophysical properties of hybrid nanofluids based on ethylene glycol with SiC nanoparticles and multi-wall carbon nanotubes (SiC-MWCNTs) were also tested for their use as coolant in automotive engine cooling systems [95]. The maximal enhancement of thermal conductivity was found to be 32.01% in hybrid nanofluids of 0.4 vol%. Bao et al. investigated hybrid water-based nanofluids containing multi-walled carbon nanotubes and SiC nanoparticles (MWCNTs-SiC) in terms of improving the photothermal conversion ability of the nanofluids [99]. Taking into account that the SiC nanofluid showed better absorption in ultraviolet (UV) and visible (VIS) light and MWCNTs in the near-IR range, the authors concluded that the hybrid nanofluids outperform one-component nanofluids in the broadband spectrum and achieve a better match with the solar spectrum.

Studies on the rheology of the water-based hybrid nanofluid ZrO_2_-SiC (ZrO_2_-SiC/W) showed that the hybrid nanofluid exhibited Newtonian properties at various temperatures and particle concentrations [96,100]. Malika and Sonawane used artificial neural network (ANN) and response surface methodology (RSM) techniques to investigate the effect of the nanoparticle mixing ratio Fe_2_O_3_:SiC on the stability and thermophysical properties of water-based hybrid nanofluid [101]. They demonstrated that the nanoparticle fraction of 20:80 vol% (Fe_2_O_3_:SiC) was the optimal mixing ratio, with improved thermophysical properties compared to the base fluid. In turn, Zhu et al. applied non-equilibrium molecular dynamics to investigate the impact of interfacial layer effects on the thermal conductivity of water-based SiC nanofluids [102]. They showed that due to the strong interaction between solid and liquid, there exists an interfacial adsorption layer with an ordered structure around the nanoparticles and that this adsorption layer is the main reason for the improvement of the heat conductivity of nanofluids. They concluded that a reduction in particle size causes an increase in the thickness of the interfacial layer, which is positively correlated with the trend of changes in thermal conductivity. In turn, Peng et al. investigated the water-based hybrid nanofluid of boron nitride nanosheets-silicon carbide (BNNs-SiC/W) in terms of use for the machining of aviation superalloys [103]. In the BNNs:SiC mass ratio of 3:2 and the particle mass concentration of 0.25%, the thermal conductivity of the hybrid nanofluid reached the highest value, 50.8% higher than the thermal conductivity of water. The viscosity of the nanofluid was 2.5% higher than the viscosity of the base fluid. Yang et al. investigated the stability of a binary nanofluid consisting of tungsten oxide (WO_2.9_) and SiC under conditions that simulate a real working environment [95]. After 20 days of cyclic thermal shock test at 90 °C, this nanofluid retained satisfactory stability. The thermal conductivity of the binary WO_2.9_-SiC nanofluid was 11.25% higher compared to the base fluid achieving a value of 0.1721 W/m·K for the WO_2.9_ (30 nm)-SiC (30 nm) nanofluid with a volume fraction of 0.13%.

It should be noted that some nanoparticles, for example, metal oxides present in nanofluids, may cause adverse effects, such as corrosion of metal components in renewable energy systems such as solar collectors [104,105]. Aluminum and copper surfaces are especially susceptible to damage by such nanofluids. Bubbico et al. [105] showed that Al_2_O_3_ and ZrO_2_ nanofluids strongly interact with aluminum, and the pH of the nanofluid has a clear effect. SiC nanofluids did not cause corrosion of aluminum. In this respect, silicon carbide nanoparticles, due to their favorable thermal properties and chemical inertness, appear to be an excellent component of nanofluids. Some studies on nanofluids fabricated with SiC nanoparticles do not provide basic information on the thermal properties and/or polytype of the silicon carbide used, which is crucial because the type of SiC crystal structure influences its physicochemical properties.

## 6. SiC Nanofluids Properties and Applications

The more interesting application of nanofluids concerns their use in photovoltaic and thermal collectors (PVTs) for the simultaneous production of heat and electricity. Numerous studies have shown an improvement in the thermal conductivity of nanofluids prepared with SiC particles, regardless of the type of base fluids used. In an earlier report, Al-Shamani et al. compared the use of various nanofluids (SiO_2_, TiO_2_ and SiC) in an experimental PVT collector under tropical climate conditions [106]. These authors demonstrated that the PVT collector with SiC nanofluid showed the highest combined PVT thermal efficiency of 81.73% and PVT electrical efficiency of 13.52% compared to other nanofluids tested or pure water. Furthermore, Al-Waeli et al. reported that a nanofluid containing 3% β-SiC nanoparticles in a hybrid PVT system caused an increase in electrical efficiency of up to 24.1% compared to the PV system alone. Moreover, the use of this nanofluid led to an increase in thermal efficiency of up to 100.19% compared to the use of water for cooling [4]. The authors also evaluated the performance of PVT collectors when working with a water-based SiC nanofluid in both indoor and outdoor systems, indicating that to ensure the stability of the thermal conductivity parameters of water-based SiC nanofluids, periodic stirring is necessary due to the sedimentation of nanoparticles [107]. Mohammadi et al. used SiC nanofluids in a mini-channel heat sink to cool electronic chipsets, showing a significant improvement in heat transfer (by 55.5%) at a concentration of SiC nanoparticles of 0.5 vol% compared to the base fluid [108]. In another report a similar application of SiC nanofluids as a cooling agent in a microchannel microfluid-coupled heat sink to cool concentrated high-power solar cells was discussed [109]. More recent studies consider the application of a specific structural type of SiC in nanofluids. Ajeena et al. observed an enhanced efficiency of a flat solar collector with β-SiC/water (β-SiC/W) nanofluid working in the conditions of the Hungarian climate [81]. The authors noted an increase in TC with a SiC nanoparticle content of 1% in the nanofluid. The maximum efficiency of the solar collector was 77.43% for 0.1% SiC/W nanofluid with a mass flow rate of 0.041 kg/s.

The flow and heat transfer characteristics of SiC/water nanofluids were also investigated at different volume fractions in microcylinder groups [110]. It was found that the coefficient of friction decreased with increasing Reynolds number. At the same time, the friction coefficient and pressure drop increased with increasing volume fraction. Due to the increasing viscosity of the SiC/water nanofluids, the Nusselt number decreased as the volume fraction of the nanoparticles increased. It was also found that increasing the volume fraction can lead to a decrease in the heat enhancement factor, showing a better heat transfer efficiency at a volume fraction of 0.2%. Karuppusamy et al. evaluated the operational efficiency of a shell and tube heat exchanger using nanofluids containing SiC nanoparticles and alkaline water (SiC/AW), as well as carbon nanotubes and alkaline water (CNT/AW) [111]. When the SiC nanofluid was compared with CNT nanofluid, the authors observed that the latter showed a noticeably reduced pressure drop, which was attributed to the improved thermophysical properties of the nanofluid. Li et al. examined a SiC nanofluid based on water and ethylene glycol mixture for use as a fluid for automotive engine cooling and concluded that increasing volume fraction and temperature can lead to improved thermal conductivity of nanofluids [112]. The highest increase in thermal conductivity (by 53.81%) was observed at 50 °C for the nanofluid with a nanoparticle concentration of 0.5 vol%. The conclusion was that due to the overall effectiveness of the coolant-based SiC nanofluid (0.2 vol%), which was found to be ~1.6, such a fluid had better application prospects compared to the regular automotive engine coolant. Koutras et al. conducted a systematic study to evaluate the influence of high temperature in situ on the loss of stability of nanofluids and their dielectric properties (TiO_2_ and SiC nanoparticles were used with natural ester oil) [113]. The main conclusion of this study was that the stability of nanofluids decreases with time, which consequently causes the agglomeration and deposition of nanoparticles. Therefore, at a given temperature, an increase in the concentration of nanoparticles caused a faster loss of stability. In this regard, SiC-containing nanofluids showed better stability than TiO_2_-prepared nanofluids.

Other research has also been conducted on the use of nanofluids containing SiC nanoparticles. The thermal performance of a hybrid photovoltaic thermal system (PVT) has been reported to be improved using nanophase change material (PCM) and water-based nanofluids, both with the addition of SiC nanoparticles [5,114]. The thermal efficiency of the heat pipes was also improved using water-based SiC nanofluids [115]. The heat removal capacity of the heat pipe increased by 29% with SiC nanofluids at a nanoparticle concentration of 1.0 wt%.

## 7. Conclusions

Current reports on nanofluids mainly focus on research on the influence of various factors on the final thermal conductivity of nanofluids. The concentration and size of nanoparticles are analyzed, as well as the grain shape and their ability to agglomerate. The influence of temperature on the viscosity of nanofluids is often discussed. As a result of its advantageous properties, SiC is one of the materials of growing interest in the preparation of nanofluids. The type of crystal structure is associated with the occurrence of certain specific characteristics of the material, such as isotropy or anisotropy. The current literature on SiC nanofluids does not explicitly examine this matter. Silicon carbide nanoparticles produced in low-temperature processes are characterized by a cubic structure and isotropic thermal conductivity. They often have a spherical morphology, which may be advantageous from the point of view of the functionality of nanoparticles in a liquid nanofluid environment. When SiC NPs are produced in a high-temperature process, anisotropic α-SiC with micro- and macroscopic grains is first produced. The larger grains are then reduced in size by grinding, but this procedure may introduce impurities into the nanometric material and thus reduce the thermal conductivity of the resulting nanoparticles. The specific nature of the grinding process may also affect the shape of the grains, leading to the formation of nanoparticles with shapes other than spherical. Hexagonal nanoparticles with heterogeneous shapes combined with their anisotropic thermal conductivity may be the reason for the discrepancies in studies of the thermal conductivity of nanofluids. Due to its high isotropic thermal conductivity, cubic β-SiC seems to be an exceptionally attractive material for the production of nanofluids. Studies on SiC nanofluids described here are difficult to compare due to differences in the type of nanoparticles used, crystal structure, grain size, particle shape, or their content in the base fluid. For this reason, in future studies, regardless of the type of structure, all nanoparticles used to produce nanofluids should be thoroughly characterized. Such detailed studies of nanoparticles will facilitate the preparation of nanofluids with properties expected for various heat transfer applications.

## Figures and Tables

**Table 1:** Lattice constants of major SiC polytypes at room temperature [53].

Polytype	*a* (nm)	*c* (nm)
3C	0.43596	-
4H	0.30798	1.00820
6H	0.30805	1.51151

**Table 2:** Thermal conductivity for the most popular SiC polytypes measured at room temperature.

SiC Polytype	Dopant Type	Measurement Direction	Measuring Method	TC [W/mK]	Comments *	Reference
3C SiC (β-SiC)	none	-	time-domain thermoreflectance TDTR	500	isotropic	[61]
4H SiC (α-SiC)	nitrogen vanadium	along C-axis	calculated from thermal diffusivity, specific heat capacity, and density	280 347	n.a.	[45]
6H SiC (α-SiC)	none	normal to C-axis parallel to C-axis	radiation thermometry	387 271	anisotropic	[62]
4H SiC (α-SiC)	nitrogen	normal to C-axis	time-domain thermoreflectance TDTR	444	anisotropic	[44]
		parallel to C-axis		302		
6H SiC (α-SiC)	vanadium	normal to C-axis		393	anisotropic	
		parallel to C-axis		273		

TC—thermal conductivity; * The nature of thermal conductivity observed by the authors.

**Table 3:** Summary of several studies on thermal conductivity of mono nanofluids prepared with silicon carbide SiC nanoparticles.

Nanoparticles	NPs Size	Base Fluid	NPs Concentrations	Thermal Conductivity Enhancement	Ref.
SiC *	~50 nm	water	0.02, 0.05 vol%	28.03% at 0.02 vol% nanoparticle concentration	[37]
SiC *; cryogenically treated SiC *	244.5 nm	water	0.1, 0.2, 0.3 wt%	13.22% (TC increase with concentration from 0.1 to 0.3 wt%) 17.56% (TC increase with concentration from 0.1 to 0.3 wt%)	
					[73]
	176.2 nm	water			
SiC *	30 nm	water/ethylene glycol (60:40)	0.2, 0.4, 0.6, 0.8, 1.0 vol%	33.84% at 1.0 vol% nanoparticle concentration	[74]

α-SiC;	20–50 nm	propylene glycol/water (30:70)	1.0, 2.0 wt%	4%	[78]
β-SiC	25 nm			11–12%	
				The authors concluded that the SiC crystal structure plays a noticeable role in increasing the thermal conductivity of the tested nanofluids. The nanomaterial with the least complex crystal structure (β-SiC, cubic) improves the thermal conductivity of the fluid in which it is dispersed more than those with the most complex crystal structure (α-SiC, hexagonal).	
β-SiC	45–65 nm	ethylene glycol (EG); propylene glycol (PG)	0.72 to 3.0 wt%	14.6% (EG); 4.8% (PG) Stability of nanofluids over 24 h. Newtonian behavior of β-SiC nanofluids for temperatures between 298.15 K and 353.15 K in the range of concentrations studied.	[79]
SiC-1 (β-SiC/Si); SiC-2 (β-SiC)	~25 nm	water	0.50, 1.0 wt%	17.62% at 1.0 wt% nanoparticle concentration; temperature 50 °C	[80]
β-SiC	45–60 nm	water	0.025, 0.05, 0.075, 0.1 vol%	30.3% at 0.1 vol% nanoparticle concentration	[81]
SiC *	40 nm	mineral oil	from 0.1 to 10.3 vol%	25% for 10.37 vol% concentration at 30 °C. An enhancement of 15.5% was observed at a concentration of 0.3 vol% in the temperature range of 30–70 °C.	[82]
α-SiC	23.85 ± 6.66 nm	ethylene glycol (EG); distilled water (DW)	0.5 to 5.0 vol%	The highest TC enhancement of 25% and 16% for SiC/EG and SiC/DW nanofluids, respectively, was observed at 28 °C and at a nanoparticle volume concentration of 5%.	[83]

NPs—nanoparticles; * polytype not specified.

**Table 4:** Summary of several studies on thermal conductivity of hybrid nanofluids prepared with silicon carbide SiC nanoparticles.

Nanoparticles	NPs Size	Base Fluid	NPs Concentrations	Thermal Conductivity Enhancement	Ref.
MWCNT: SiC * (1:9)	MWCNT: 20 nm; SiC: 40 nm	thermal oil with oleic acid (various concentrations)	0.05, 0.1, 1.0 vol%	22.58% at a nanoparticle concentration of 1.0 vol% and a temperature of 80 °C. In terms of viscosity, the optimal concentration of nanoparticles was 0.1% vol% with an oleic acid content in thermal oil of 5%.	[93]
MWCNT-SiC * (8:2)	MWCNT: 20 nm; SiC: 40 nm	ethylene glycol	0.04, 0.1, 0.2, 0.4 vol%	32.01% at a nanoparticle concentration of 0.4 vol%	[94]
SiC *:Al_2_O_3_	SiC: ~50 nm;	water	0.02 vol% and 0.05 vol%	13.10% at room temperature; an increase of 20.06% was recorded after increasing the nanoparticle concentration to 0.05 vol%. 14.92% at room temperature and at a nanoparticle concentration of 0.02 vol%	[39]
	Al_2_O_3_: (#)				
SiC *:SiO_2_	SiC: ~50 nm;				
	SiO_2_: (#)				
SiC *:WO_2.9_	30 nm	heat transfer oil	0.01, 0.05, 0.09, 0.13, 0.17, 0.21, 0.25 vol%	11.25% at nanoparticles concentration of 0.13 vol%; the homogeneous nanofluid prepared from chemically modified nanoparticles maintained good stability after 20 days of cyclic thermal shock experiment at 90 °C.	[95]
β-SiC:ZrO_2_ (1:1)	SiC: 45–60 nm; ZrO_2_: 20 nm	distilled water	0.025, 0.05, 0.075, 0.1 vol%	25.75% at 60 °C and at nanoparticle concentration of 0.1 vol%	[96]

NPs—nanoparticles; * polytype not specified; (#) NPs size not specified.

## Data Availability

No new data were created or analyzed in this study.
